# Safety and efficacy of empagliflozin in heart failure among patients with a history of valvular heart disease: Insights from EMPEROR-Pooled

**DOI:** 10.1016/j.xjon.2025.03.018

**Published:** 2025-04-01

**Authors:** Nitish K. Dhingra, Ekene Nwajei, Raj Verma, Egon Pfarr, Tomasz Gasior, Subodh Verma

**Affiliations:** a Division of Cardiac Surgery, St Michael's Hospital, Toronto, Ontario, Canada; b Boehringer Ingelheim International GmbH, Ingelheim, Germany; c Collegium Medicum, Faculty of Medicine, WSB University, Dabrowa Gornicza, Poland

**Keywords:** co-transporter 2 inhibitors, heart failure, sodium-glucose, valvular heart disease

## Abstract

**Background:**

Valvular heart disease (VHD)-associated heart failure (HF) remains an important and growing cause of morbidity and mortality. There are no contemporary data on the efficacy and safety of SGLT2 inhibitors in patients with a history of VHD.

**Methods:**

The EMPEROR-Pooled trial analyzed 9718 patients with HF who were enrolled in the randomized trials of empagliflozin versus placebo in HF with reduced left ventricular ejection fraction (HfrEF; EMPEROR-Reduced) and HF with preserved left ventricular ejection fraction (HFpEF; EMPEROR-Preserved). These trials evaluated a primary outcome of time to first HF hospitalization or cardiovascular death. Here we analyze outcomes of the EMPEROR-Pooled patients according to the presence and etiology of VHD history.

**Results:**

Of the 9717 patients enrolled in EMPEROR-Pooled with available data, 1484 (15.3%) had a history of VHD. Of the patients with VHD history, a history of isolated mitral disease (39.2%) was the most common subtype. In patients randomized to placebo, the risk of the primary outcome was higher among patients with VHD history (hazard ratio [HR], 1.30; 95% confidence interval [CI], 1.10-1.53; *P* < .01), and particularly those with a history of multivalvular disease (HR, 1.51; 95% CI, 1.13-2.04; *P* < .01) compared with no valvular disease. No heterogeneity was introduced by VHD history with respect to the efficacy of empagliflozin on all major clinical outcomes evaluated in EMPEROR-Pooled (*P*_interaction_ > .05).

**Conclusions:**

We present the first large analysis of SGLT2i (empagliflozin) use in HF patients by history of VHD. Although VHD history was associated with worse outcomes in HF patients, empagliflozin demonstrated consistent safety, efficacy, and patient-reported outcomes across all categories of VHD history.

**Figure:**
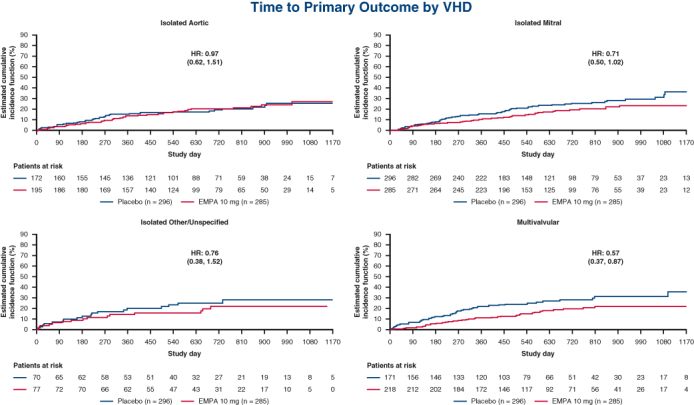
Efficacy of empagliflozin stratified by VHD history subtype.

**Central Message**

The efficacy of empagliflozin in patients with heart failure is maintained irrespective of valvular heart disease history, despite an elevated baseline risk of clinical outcomes in this subgroup.

**Perspective**

In the EMPEROR-Pooled trial, patients with a valvular heart disease (VHD) history, and particularly those with a history of multivalvular disease, had an elevated risk of clinical outcomes compared to patients without a VHD history. Nevertheless, the efficacy and safety of empagliflozin was consistent regardless of the presence or etiology of VHD history.

Lesions of cardiac valvular structures represent an important cause of clinical heart failure (HF), second only to ischemic heart disease as a central etiology.^1^ Beyond the incidence and prevalence of valvular heart disease (VHD)-related HF, outcomes of patients with VHD and heart failure are significantly worse than those without VHD. Indeed, for the primary outcome of all-cause mortality or rehospitalization, a cluster analysis of nearly 1700 patients hospitalized with HF demonstrated a hazard ratio (HR) of 1.88 and 1.70 associated with VHD at 6 months and 12 months, respectively.^2^ Despite the scope of this issue, evidence to inform the medical management of patients with VHD remains limited.^3^^,^^4^

Empagliflozin, a sodium-glucose co-transporter 2 (SGLT2) inhibitor, has recently emerged as an efficacious pharmacotherapeutic agent in improving both clinical and patient-reported outcomes for HF with reduced, mid-range, and preserved left ventricular ejection fraction (LVEF: HFrEF, HFmrEF, and HFpEF, respectively).^5^^,^^6^ To determine whether the safety and efficacy of this agent was maintained among patients with VHD, this post hoc subgroup analysis evaluated outcomes with empagliflozin in the EMPEROR program, stratified by the presence and etiology of VHD history.

## Methods

### Trial Design

The EMPEROR-Reduced and EMPEROR-Preserved trials randomized patients with chronic HF to empagliflozin 10 mg/day versus placebo. The specific methods of these trials have been published previously.^5^^,^^6^ In summary, the EMPEROR program included adults with chronic heart failure (New York Heart Association class II-IV) along with natriuretic peptide elevation. The trial in which participants were randomized depended on their LVEF; those with HFrEF (LVEF <40%) were randomized in EMPEROR-Reduced, and those with HFmrEF or HFpEF (LVEF ≥40%) were randomized in EMPEROR-Preserved. Either evidence of structural heart disease or hospitalization for HF in the previous 12 months also was required for eligibility in EMPEROR-Preserved. An estimated glomerular filtration rate (eGFR) <20 mL/min/1.73 m^2^ and hypotension (symptomatic and/or systolic blood pressure <100 mm Hg) were exclusionary factors. EMPEROR-Pooled was a prospectively designed combined analysis of these landmark trials. Each trial center received Institutional Review Board approval, and informed consent was mandatory for every participating patient.

### Study Group Formation

Patients were identified at baseline as having a history of clinically meaningful valvular heart disease, with enrolling clinicians being prompted to provide further details regarding the nature of VHD. Based on these entries, patients were manually assigned to 1 of 5 categories by the coauthors, independent of Boehringer Ingelheim (BI), based on blinded treatment group info: history of isolated aortic disease, isolated mitral disease, isolated other/unspecified VHD, multivalvular disease, and no VHD.

### Study Outcomes

The index trials evaluated a primary outcome of time to first HF hospitalization or cardiovascular (CV) death. In the present analysis, secondary outcomes assessed included time to first HF hospitalization, total (first and recurrent) HF hospitalizations, time to CV death, and time to all-cause mortality. The slope of eGFR change, as well as Kansas City Cardiomyopathy Questionnaire clinical summary scores (KCCQ-CSS) also were evaluated in this subgroup analysis. Finally, a safety analysis was conducted, with descriptive statistics collected for specific adverse events, including total adverse events, serious adverse events, and adverse events leading to treatment discontinuation, as well as acute renal failure, volume depletion, hypotension, hyperkalemia, genital infection, confirmed hypoglycemia, bone fracture, and ketoacidosis.

### Statistical Analysis

Time-to-first event analyses were conducted using a Cox proportional hazards model, with adjustment made for age, sex, study, diabetes status (diabetes, prediabetes, no diabetes), eGFR at baseline, LVEF at baseline, and region (North America, Latin America, Europe, Asia, and other). The adjusted HRs are reported here. A joint frailty model that accounted for CV death was used to evaluate total (first and recurrent) hospitalization for heart failure (HHF), with adjustments made for the same covariates as for the Cox model. These covariates were similarly adjusted for in the evaluation of continuous endpoints in a mixed model with repeated measures, along with adjustment for visit by treatment by VHD history interaction and baseline value by visit interaction. The consistency of treatment effects across VHD subgroups was evaluated using subgroup-by-treatment interaction terms. The intention-to-treat principle was used for all analyses, and data up to the end of the planned treatment period were included. Descriptive statistics were used to assess rates of adverse events occurring during the on-treatment period (including 7 days after the last drug consumption).

## Results

Among the 9717 patients with available data from EMPEROR-Pooled, the majority (n = 8233; 84.7%) did not have a documented history of VHD. Of the 1484 patients (15.3%) with a history of VHD, 367 (24.7%) had a history of isolated aortic valve disease, 581 (39.2%) had a history of isolated mitral valve disease, 389 (26.2%) had a history of multivalvular disease, and 147 (9.91%) had a history of isolated other/unspecific VHD. Baseline characteristics of patients enrolled in EMPEROR-Pooled stratified by the presence and etiology of VHD history are presented in Table E1.

Among patients randomized to placebo, those with VHD history, and specifically those with a multivalvular disease history, were at elevated risk of HF outcomes in EMPEROR-Pooled (Table 1). Compared to patients with no VHD history, those with a VHD history had an elevated risk of the primary outcome (HR, 1.30; 95% CI, 1.10-1.53; *P* < .01), total HHF (HR, 1.41; 95% CI, 1.11-1.78; *P* < .01), time to first HHF (HR, 1.27; 95% CI, 1.04-1.55; *P* = .02), and time to all-cause mortality (HR, 1.29; 95% CI, 1.05-1.57; *P* = .01), with no statistical difference in time to CV death. These associations were magnified in patients with a multivalvular disease history for the primary outcome (HR, 1.51; 95% CI, 1.13-2.04; *P* < .01), time to first HHF (HR, 1.60; 95% CI, 1.15-2.24; *P* < .01), and total (first and recurrent) HHF (HR, 1.61; 95% CI, 1.04-2.48; *P* = .03). Similar trends were noted for patients with isolated other/unspecified VHD history, although reaching significance only for total (first and recurrent) HHF (HR, 2.54; 95% CI, 1.30-4.95; *P* < .01) and approaching significance for time to CV death. Patients with isolated mitral disease history were at higher risk of mortality in EMPEROR-Pooled (time to all-cause mortality: HR, 1.39; 95% CI, 1.04-1.84; *P* = .02), albeit with no statistically significant elevations in risk for the remaining clinical outcomes. Patients randomized to placebo with isolated aortic disease history and patients without a VHD history had a comparable risk of clinical outcomes in this analysis (*P* > .25 for all outcomes). Comparable slopes of eGFR were documented across VHD history subgroups (data not shown).

**Table 1:** Risk of HF outcomes in EMPEROR-Pooled according to presence and etiology of VHD history among patients randomized to placebo

Group	HR (95% CI)∗	*P* value
Time to first event of HHF or CV death		
Any VHD	1.30 (1.10-1.53)	<.01
Isolated aortic	1.17 (0.84-1.64)	.35
Isolated mitral	1.22 (0.95-1.55)	.12
Isolated other/unspecified	1.48 (0.92-2.36)	.10
Multivalvular	1.51 (1.13-2.04)	<.01
Total (first and recurrent) HHF		
Any VHD	1.41 (1.11-1.78)	<.01
Isolated aortic	1.13 (0.71-1.80)	.60
Isolated mitral	1.24 (0.88-1.76)	.22
Isolated other/unspecified	2.54 (1.30-4.95)	<.01
Multivalvular	1.61 (1.04-2.48)	.03
Time to first HHF		
Any VHD	1.27 (1.04-1.55)	.02
Isolated aortic	1.13 (0.76-1.68)	.55
Isolated mitral	1.13 (0.84-1.51)	.42
Isolated other/unspecified	1.51 (0.87-2.63)	.14
Multivalvular	1.60 (1.15-2.24)	<.01
Time to CV death		
Any VHD	1.22 (0.94-1.57)	.13
Isolated aortic	1.02 (0.61-1.72)	.94
Isolated mitral	1.18 (0.82-1.71)	.37
Isolated other/unspecified	1.79 (0.95-3.37)	.07
Multivalvular	1.25 (0.79-2.00)	.34
Time to all-cause mortality		
Any VHD	1.29 (1.05-1.57)	.01
Isolated aortic	1.22 (0.84-1.77)	.29
Isolated mitral	1.39 (1.04-1.84)	.02
Isolated other/unspecified	1.53 (0.88-2.65)	.13
Multivalvular	1.09 (0.73-1.63)	.67

*HF*, Heart failure; *VHD*, valvular heart disease; *HR*, hazard ratio; *CI*, confidence interval; *HHF*, hospitalization for heart failure; *CV*, cardiovascular.

∗ Comparison group: no VHD.

**Figure 1:**
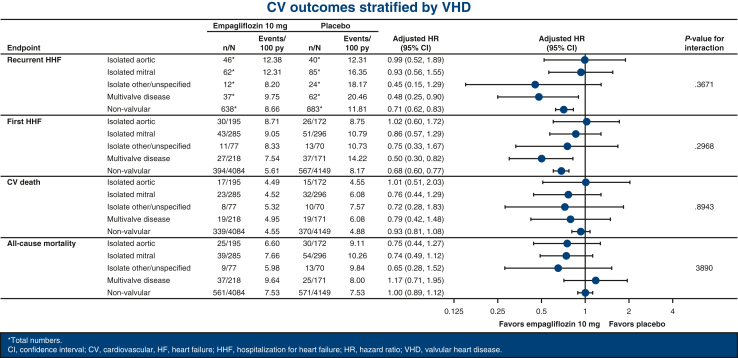
Rates of secondary outcomes in patients randomized to empagliflozin versus placebo stratified by valvular heart disease (*VHD*) history.

**Figure E1:**
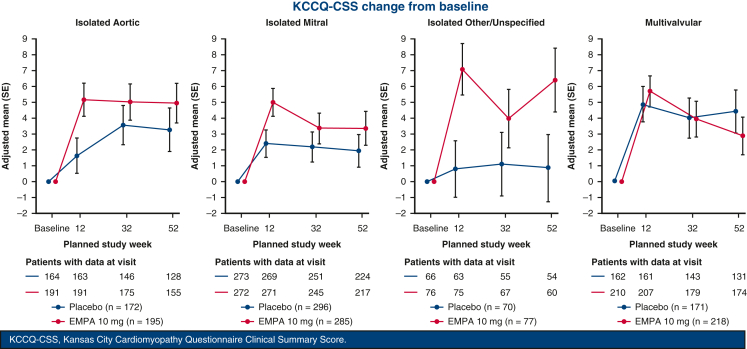
Change in Kansas City Cardiomyopathy Questionnaire Clinical Summary Score (*KCCQ-CSS*) from baseline to the 52-week follow-up with empagliflozin (*EMPA*) versus placebo stratified by valvular heart disease history.

Despite this elevated risk, the efficacy of empagliflozin in reducing the risk of HF outcomes in EMPEROR-Pooled was entirely consistent irrespective of VHD history. Indeed, comparable HRs were documented between patients with any VHD history (HR, 0.73; 95% CI, 0.59-0.91) and those with no VHD (0.77; 95% CI, 0.70-0.86; *P*_interaction_ = .66) and within each valvular subgroup for the primary outcome (aortic: HR, 0.97 [95% CI, 0.62-1.51]; mitral: HR, 0.71 [95% CI, 0.50-1.02]; other/unspecified: HR, 0.76 [95% CI, 0.38-1.52]; multivalvular: HR, 0.57 [95% CI, 0.37-0.87]; no VHD: HR, 0.77 [95% CI, 0.70-0.86]; *P*_interaction_ = .54). Similarly, there were no significant interactions of VHD history on the efficacy of empagliflozin as it pertained to total (first and recurrent) HHF, time to first HHF, time to CV death, and time to all-cause mortality (Figure 1). Finally, no heterogeneity was documented for adjusted mean change from baseline in KCCQ-CSS at 52 weeks (*P*_interaction_ = .62 between VHD history and no VHD history and .30 across valvular subgroups) (Figure E1) and for slope of eGFR (*P*_interaction_ = .32 between VHD history and no VHD history and .53 across valvular subgroups).

With respect to safety outcomes, patients with VHD history, regardless of randomized intervention, tended to have higher rates of any adverse effects (AEs) (any VHD: placebo, 88.1%, empagliflozin, 85.2%; aortic: placebo, 86.0%; empagliflozin, 85.6%; mitral: placebo, 88.8%; empagliflozin. 82.5%; other/unspecified: placebo, 87.1%; empagliflozin, 83.1%; multivalvular: placebo, 89.4%; empagliflozin, 89.0%; no VHD: placebo, 82.6%; empagliflozin, 81.6%) and serious AEs (any VHD: placebo, 58.1%; empagliflozin, 50.7%; aortic: placebo, 57.0%; empagliflozin, 51.8%; mitral: placebo, 54.9%; empagliflozin, 48.1%; other/unspecified: placebo, 65.7%; empagliflozin, 46.8%; multivalvular: placebo, 61.8%; empagliflozin, 54.6%; no VHD: placebo, 48.9%; empagliflozin, 44.5%). Nevertheless, the safety profile of empagliflozin was similar irrespective of VHD history, including for AEs of interest, such as acute renal failure, volume depletion, hypotension, and hyperkalemia (Table E2).

## Discussion

This subanalysis of EMPEROR-Pooled investigated the impact of a history of VHD on clinical outcomes in a contemporary cohort of adult patients with HF and also assessed for any heterogeneity introduced by VHD history to the efficacy and safety of empagliflozin in HF patients. To this end, we report three central findings. First, in EMPEROR-Pooled, patients with VHD history, particularly those with a history of multivalvular disease, were at an elevated risk of HF outcomes compared with patients without a history of VHD. Second, despite this elevated baseline risk, the existence and etiology of VHD history did not alter the efficacy of empagliflozin. Finally, while patients with VHD history tended to have numerically higher rates of AEs regardless of randomized therapy, the safety profile of empagliflozin was unaffected by VHD history in EMPEROR-Pooled.

The relationship between VHD and HF has been well described. Indeed, HF represents an inevitable consequence of the natural history of VHD, secondary to alterations in loading conditions, cardiac remodeling, and impairment of diastolic and/or systolic function. According to multiple international registries, VHD represents a central etiology in 11.8% to 28.1% of patients with HF.^1^ In the Euro Heart Survey, which prospectively enrolled more than over 5000 adults with moderate-severe VHD from 25 countries in the early 2000s, isolated aortic stenosis and mitral regurgitation (MR) were the most common valvular lesions documented.^7^ Importantly, however, not only can VHD be an important etiology of HF, but also valvular lesions may be further exacerbated by the vicious cycle introduced by progressive left ventricular (LV) systolic dysfunction, worsening congestion, and consequent ventricular remodeling. For example, secondary MR develops as a result of annular dilatation from progressive LV dysfunction in the setting of a normal mitral valve.^8^^,^^9^ Secondary MR represents the most common source of MR^10^ and has significant prognostic implications. In a meta-analysis of 53 studies encapsulating 45,900 patients, secondary MR was associated with significantly increased risks of all-cause mortality even when mild in degree and also was associated with increased risks of HHF, CV death, and death, HF, and transplant.^11^ In our analysis, patients with isolated mitral valve disease history were the only subgroup of patients with VHD to show an elevated risk of mortality compared with non-VHD patients.

Although structural interventions, ranging from percutaneous approaches to surgical operations, remain central components in management of VHD-related HF, finding evidence-based pharmacotherapies to aid the treatment this patient population remains a clinical priority. In the case of aortic stenosis, medical therapy is currently limited in its ability to prevent progression of disease and thus is generally used for symptom benefit and afterload reduction.^4^ It is notable that despite this, patients with isolated aortic disease history had a comparable efficacy of empagliflozin on hard clinical outcomes as patients with no VHD or other etiologies of VHD in EMPEROR-Pooled.

However, for MR, particularly secondary MR, medical therapy has a more central role in preventing the aforementioned vicious cycle triggered by LV dilatation and systolic dysfunction. Guideline-directed medical therapy for HFrEF, including therapy with beta-blockers^12^ and angiotensin receptor-neprilysin inhibition (ARNI),^13^ has been shown to have a favorable impact on secondary MR associated with clinical HF through reducing LV end-diastolic volumes and regurgitant volumes/MR severity. Prospective studies have demonstrated the ability of guideline-directed medical therapy to resolve severe MR in nearly 40% of patients with HFrEF-associated secondary MR, with consequent improvements in prognosis as well.^14^ In EMPEROR-Pooled, despite the background elevated risk of mortality, patients with isolated mitral valve disease history randomized to empagliflozin were noted to have lower HRs with respect to the primary outcome, CV death, and all-cause mortality compared with patients without VHD, albeit with no statistically significant heterogeneity.

These effects can be explained by previous findings that although SGLT2 inhibitors like empagliflozin are not directly cardioactive, they may induce cardiac remodeling in at-risk patients. For example, randomized data have shown significant reductions in indexed LV mass noted after 6 months of treatment with empagliflozin in patients with type 2 diabetes mellitus and coronary artery disease, although without any improvement in LV end-diastolic volume.^15^ These cardiac effects may be a result of improved myocardial oxygen delivery and cardiomyocyte mitochondrial function secondary to enhanced erythropoiesis,^15^, ^16^, ^17^, ^18^ autophagic clearance of organelles and consequent decreased cellular stress and inflammation mediated by upregulation of nutrient deprivation signaling,^19^, ^20^, ^21^, ^22^, ^23^ as well as osmotic diuresis leading to decreased ventricular congestion.^24^, ^25^, ^26^, ^27^, ^28^, ^29^, ^30^, ^31^

Although the present findings corroborate previous subanalyses from the EMPEROR trials demonstrating the wide generalizability of empagliflozin across a variety of clinically relevant subgroups,^32^, ^33^, ^34^, ^35^, ^36^, ^37^ this investigation has some inherent limitations that should be considered. First, to ensure adequate volumes of patients in each subgroup to enable meaningful comparisons, relatively broad categories of VHD were formulated. It is clear that patients with, for example, MR and mitral stenosis may have important differences in ventricular loading conditions and hemodynamics and may respond differently to SGLT2 inhibition. Second, the case report form did not include a systematic gathering of information related to the severity of valvular lesions and whether intervention for the lesion had already been conducted. As such, we did not require uncorrected valvular lesions or define a minimum severity of valvular lesions for inclusion into the VHD subgroups, which may have biased the results toward the null. Finally, this investigation was post hoc, with no corrections for multiplicity, and thus its findings are hypothesis-generating and warrant further prospective study for confirmation. Nevertheless, this paper represents the first large analysis of SGLT2i use in people with VHD history and HF using prospective and randomized data.

In summary, although patients with a VHD history, particularly those with multivalvular disease history, experienced a higher risk of clinical HF outcomes compared with their counterparts with no VHD history in EMPEROR-Pooled, empagliflozin demonstrated consistent efficacy and safety in HF patients irrespective of the presence and etiology of VHD history.

## Conflict of Interest Statement

N.K.D. reported statistical support from Amarin, Boehringer Ingelheim, Eli Lilly, Lexicon Pharmaceuticals, and Sanofi. E.P. and T.G. are employees of Boehringer Ingelheim. S.V. is supported by the Canadian Institutes of Health Research and Heart and Stroke Foundation of Canada; holds the Tier 1 Canada Research Chair in Cardiovascular Surgery; and has received speaking honoraria and/or consulting fees from Abbott, Amarin, AstraZeneca, Bayer, Boehringer Ingelheim, Canadian Medical and Surgical Knowledge Translation Research Group, Eli Lilly, HLS Therapeutics, Janssen, Merck, Novartis, Novo Nordisk, Pfizer, PhaseBio, and TIMI. All other authors reported no conflicts of interest.

The *Journal* policy requires editors and reviewers to disclose conflicts of interest and to decline handling or reviewing manuscripts for which they may have a conflict of interest. The editors and reviewers of this article have no conflicts of interest.

## Figures and Tables

**Table E1:** Background characteristics of patients enrolled in EMPEROR-Pooled stratified by the presence and etiology of VHD history

Characteristic	Any VHD (N = 1484)	Isolated aortic (N = 367)	Isolated mitral (N = 581)	Isolated other/unspecified (N = 147)	Multivalvular (N = 389)	No VHD (N = 8233)
Demographics						
Male sex, n (%)	894 (60.2)	241 (65.7)	355 (61.1)	71 (48.3)	227 (58.4)	5254 (63.8)
White race, n (%)	1113 (75.0)	294 (80.1)	432 (74.4)	106 (72.1)	281 (72.2)	6057 (73.6)
Age, y, mean (SD)	71.2 (10.4)	73.7 (9.2)	69.4 (10.6)	70.0 (11.4)	72.3 (10.3)	69.7 (10.3)
Cardiac risk factors						
Smoking history, n (%)	732 (49.3)	197 (53.7)	291 (50.1)	57 (38.8)	187 (48.1)	4146 (50.4)
Hypertension, n (%)	1220 (82.2)	316 (86.1)	465 (80.0)	118 (80.3)	321 (82.5)	6901 (83.8)
Hypercholesterolemia, n (%)	993 (66.9)	273 (74.4)	397 (68.3)	80 (54.4)	243 (62.5)	5300 (64.4)
Diabetes, n (%)	601 (40.5)	157 (42.8)	239 (41.1)	52 (35.4)	153 (39.3)	4192 (50.9)
BMI, kg/m^2^, mean (SD)	28.10 (5.66)	29.29 (5.57)	27.95 (5.56)	27.91 (5.67)	27.25 (5.72)	29.26 (5.77)
Body weight, kg, mean (SD)	77.93 (18.98)	82.27 (18.81)	77.06 (18.34)	76.92 (18.82)	75.50 (19.54)	81.13 (19.13)
HF characteristics and established cardiovascular disease						
LVEF, %, mean (SD)	43.9 (16.0)	48.4 (14.7)	40.2 (15.8)	49.1 (16.6)	43.2 (15.6)	44.0 (15.1)
NT-proBNP, pg/mL, median (IQR)	1549 (842.50- 2589.50)	1305 (677- 2494)	1605 (908- 2557)	1347 (807- 2338)	1676 (1057- 3160)	1242 (642, 2273)
Atrial fibrillation or flutter, n (%)∗	926 (62.4)	208 (56.7)	347 (59.7)	99 (67.3)	272 (69.9)	3649 (44.3)
History of HHF in previous 12 mo, n (%)†	419 (28.2)	87 (23.7)	169 (29.1)	46 (31.3)	117 (30.1)	2101 (25.5)
Etiology of HF, n (%)						
Ischemic	502 (33.8)	108 (29.4)	243 (41.8)	30 (20.4)	121 (31.1)	3543 (43.0)
Hypertensive	279 (18.8)	82 (22.3)	88 (15.1)	35 (23.8)	74 (19.0)	2360 (28.7)
VHD	429 (28.9)	127 (34.6)	148 (25.5)	47 (32.0)	107 (27.5)	31 (0.4)
Diabetes	5 (0.3)	0	3 (0.5)	0	2 (0.5)	167 (2.0)
Alcoholism	6 (0.4)	0	2 (0.3)	1 (0.7)	3 (0.8)	48 (0.6)
Idiopathic	93 (6.3)	19 (5.2)	29 (5.0)	15 (10.2)	30 (7.7)	1095 (13.3)
Other	170 (11.5)	31 (8.4)	68 (11.7)	19 (12.9)	52 (13.4)	988 (12.0)
KCCQ-CSS, mean (SD)						
NYHA class, n (%)						
I	1 (0.1)	0	0	1 (0.7)	0	3 (<0.1)
II	1065 (71.8)	271 (73.8)	429 (73.8)	95 (64.6)	270 (69.4)	6617 (80.4)
III	407 (27.4)	94 (25.6)	150 (25.8)	47 (32.0)	116 (29.8)	1586 (19.3)
IV	11 (0.7)	2 (0.5)	2 (0.3)	4 (2.7)	3 (0.8)	27 (0.3)
Time since diagnosis of HF, y, mean (SD)	5.8 (6.1)	5.3 (6.0)	6.2 (6.2)	5.8 (6.7)	5.5 (5.6)	4.9 (5.6)
Euvolemia in the previous 4 wk, n (%)	782 (52.7)	188 (51.2)	303 (52.2)	73 (49.7)	218 (56.0)	4715 (57.3)
Myocardial infarction, n (%)	454 (30.6)	99 (27.0)	219 (37.7)	29 (19.7)	107 (27.5)	2948 (35.8)
Coronary artery disease, n (%)	598 (40.3)	169 (46.0)	256 (44.1)	35 (23.8)	138 (35.5)	3205 (38.9)
Invasive electrophysiologic procedure, n (%)	145 (9.8)	34 (9.3)	61 (10.5)	6 (4.1)	44 (11.3)	518 (6.3)
Electrical cardioversion in previous 6 mo, n (%)	38 (2.6)	12 (3.3)	9 (1.5)	5 (3.4)	12 (3.1)	129 (1.6)
Other clinically important arrythmias, n (%)	235 (15.8)	42 (11.4)	106 (18.2)	22 (15.0)	65 (16.7)	808 (9.8)
Laboratory values and background therapies						
eGFR, mL/min/1.73 m^2^, mean (SD)	58.9 (20.5)	57.0 (20.1)	59.9 (20.3)	60.0 (20.0)	58.8 (21.2)	61.6 (20.5)
Hemoglobin, g/dL, mean (SD)	13.22 (1.57)	13.20 (1.55)	13.35 (1.61)	13.16 (1.5)	13.07 (1.56)	13.51 (1.61)
Background therapies, n (%)						
ACEi/ARB/ARNI	1164 (78.4)	276 (75.2)	470 (80.9)	107 (72.8)	311 (79.9)	6960 (84.5)
Beta-blockers	1328 (89.5)	314 (85.6)	527 (90.7)	132 (89.8)	355 (91.3)	7371 (89.5)
MRA	783 (52.8)	151 (41.1)	344 (59.2)	76 (51.7)	212 (54.5)	4121 (50.1)
Loop or high ceiling diuretics	1192 (80.3)	273 (74.4)	478 (82.3)	129 (87.8)	312 (80.2)	6011 (73.0)
ACEi/ARB/ARNI + beta-blocker/ivabradine + MRA	601 (40.5)	107 (29.2)	271 (46.6)	54 (36.7)	169 (43.4)	3330 (40.4)
Other antihypertensives	287 (19.3)	88 (24.0)	97 (16.7)	28 (19.0)	74 (19.0)	1821 (22.1)
Lipid-lowering therapies	1018 (68.6)	279 (76.0)	412 (70.9)	78 (53.1)	249 (64.0)	5836 (70.9)
Antiplatelet therapies	623 (42.0)	168 (45.8)	257 (44.2)	51 (34.7)	147 (37.8)	4204 (51.1)
Anticoagulation	916 (61.7)	207 (56.4)	364 (62.7)	91 (61.9)	254 (65.3)	3454 (42.0)
ICD or CRT-D, n (%)	275 (18.5)	49 (13.4)	143 (24.6)	13 (8.8)	70 (18.0)	1128 (13.7)
CRT or non-CRT pacemaker, n (%)	236 (15.9)	68 (18.5)	93 (16.0)	17 (11.6)	58 (14.9)	765 (9.3)

*VHD*, Valvular heart disease; *BMI*, body mass index; *HF*, heart failure; *LVEF*, left ventricular ejection fraction; *NT-proBNP*, N-terminal pro B-type natriuretic peptide; *IQR*, interquartile range; *HHF*, hospitalization for heart failure; *KCCQ-CSS*, Kansas City Cardiomyopathy Questionnaire Clinical Summary Score; *NYHA*, New York Heart Association; *eGFR*, estimated glomerular filtration rate; *ACEi*, angiotensin-converting enzyme inhibitors; *ARB*, angiotensin II receptor blockers; *ARNI*, angiotensin receptor-neprilysin inhibition; *MRA*, mineralocorticoid receptor antagonists; *ICD*, implantable cardioverter-defibrillator; *CRT*, cardiac resynchronization therapy.

∗ Defined as atrial fibrillation or atrial flutter revealed on any electrocardiogram before atrial fibrillation or atrial flutter reported in the medical history.

† Reported either on HF history and diagnosis or Health Care Resource Utilization form.

**Table E2:** AEs in the EMPEROR program stratified by presence and etiology of VHD history

AE	Any VHD (N = 1484)		Isolated aortic (N = 367)		Isolated mitral (N = 581)		Isolated other/unspecified (N = 147)		Multivalvular (N = 389)		No VHD (N = 8233)	
	Placebo (N = 707)	Empa (N = 775)	Placebo (N = 172)	Empa (N = 195)	Placebo (N = 295)	Empa (N = 285)	Placebo (N = 70)	Empa (N = 77)	Placebo (N = 170)	Empa (N = 218)	Placebo (N = 4145)	Empa (N = 4083)
Total with any AE, n (%)	623 (88.1)	660 (85.2)	148 (86.0)	167 (85.6)	262 (88.8)	235 (82.5)	61 (87.1)	64 (83.1)	152 (89.4)	194 (89.0)	3425 (82.6)	3333 (81.6)
Total with serious AE, n (%)	411 (58.1)	393 (50.7)	98 (57.0)	101 (51.8)	162 (54.9)	137 (48.1)	46 (65.7)	36 (46.8)	105 (61.8)	119 (54.6)	2028 (48.9)	1815 (44.5)
Total with AEs leading to treatment discontinuation, n (%)	140 (19.8)	164 (21.2)	34 (19.8)	41 (21.0)	59 (20.0)	59 (20.7)	16 (22.9)	17 (22.1)	31 (18.2)	47 (21.6)	739 (17.8)	729 (17.9)
Acute renal failure, n (%)	102 (14.4)	86 (11.1)	27 (15.7)	18 (9.2)	42 (14.2)	30 (10.5)	12 (17.1)	12 (15.6)	21 (12.4)	26 (11.9)	474 (11.4)	452 (11.1)
Volume depletion, n (%)	86 (12.2)	99 (12.8)	18 (10.5)	21 (10.8)	32 (10.8)	30 (10.5)	8 (11.4)	10 (13.0)	28 (16.5)	38 (17.4)	384 (9.3)	454 (11.1)
Hypotension, n (%)	79 (11.2)	88 (11.4)	18 (10.5)	18 (9.2)	28 (9.5)	29 (10.2)	7 (10.0)	8 (10.4)	26 (15.3)	33 (15.1)	341 (8.2)	399 (9.8)
Hyperkalemia, n (%)	53 (7.5)	42 (5.4)	13 (7.6)	12 (6.2)	23 (7.8)	19 (6.7)	7 (10.0)	4 (5.2)	10 (5.9)	7 (3.2)	270 (6.5)	238 (5.8)
Genital infection, n (%)	2 (0.3)	14 (1.8)	0	3 (1.5)	1 (0.3)	9 (3.2)	0	1 (1.3)	1 (0.6)	1 (0.5)	32 (0.8)	84 (2.1)
Confirmed hypoglycemia, n (%)∗	10 (1.4)	9 (1.2)	5 (2.9)	2 (1.0)	3 (1.0)	4 (1.4)	1 (1.4)	0	1 (0.6)	3 (1.4)	96 (2.3)	91 (2.2)
Bone fracture, n (%)	30 (4.2)	27 (3.5)	6 (3.5)	4 (2.1)	12 (4.1)	9 (3.2)	6 (8.6)	3 (3.9)	6 (3.5)	11 (5.0)	138 (3.3)	152 (3.7)
Ketoacidosis, n (%)	0	1 (0.1)	0	0	0	0	0	0	0	1 (0.5)	5 (0.1)	3 (0.1)

*AE*, Adverse event; *VHD*, valvular heart disease; *Empa*, empagliflozin.

∗ Hypoglycemic AEs with plasma glucose ≤70 mg/dL or requiring assistance.
